# Impact of novel software on laboratory expenditure at an academic hospital in South Africa

**DOI:** 10.4102/ajlm.v12i1.2159

**Published:** 2023-11-30

**Authors:** Zoliswa Mayekiso, Kelechi E. Oladimeji, Guillermo A. Pulido Estrada, Charles Hongoro, Teke R. Apalata

**Affiliations:** 1 Department of Laboratory Medicine & Pathology, Faculty of Health Sciences, Walter Sisulu University, Mthatha, South Africa; 2 Department of Public Health, Faculty of Health Sciences, Walter Sisulu University, Mthatha, South Africa; 3 Ezintsha, Faculty of Health Sciences, University of the Witwatersrand, Johannesburg, South Africa; 4 Developmental, Capable and Ethical State Division, Human Sciences Research Council (HSRC), Pretoria, South Africa; 5 School of Health Systems and Public Health, University of Pretoria, Pretoria, South Africa

**Keywords:** Electronic gatekeeping, medical laboratory expenditure, interrupted time series analysis, cost-effectiveness, intervention, rural, academic hospital, South Africa

## Abstract

**Background:**

Countries across the globe report an increase in expenditure associated with medical laboratory testing. In 2020, the United States Department of Health and Human Services reported that laboratory test expenditures increased by $459 million US dollars (USD) from $7.1 billion USD in 2018. In South Africa, laboratory testing expenditure in the public sector increased from $415 million USD in 2014 to $723 million USD in 2021.

**Objective:**

This study aimed to evaluate the impact of an innovative software, electronic gatekeeping (EGK), on medical laboratory test expenditures at Nelson Mandela Academic Hospital, in the Eastern Cape, South Africa.

**Methods:**

In this cross-sectional study, an interrupted time series analysis technique was used to evaluate trends in expenditure during a 48-month study period. To measure the impact of EGK on laboratory expenditure, we analysed laboratory expenditure over two study periods: a period of 24 months occurring before EGK implementation (01 June 2013 to 31 May 2015) and a period of 24 months occurring during EGK implementation (01 June 2015 to 30 May 2017).

**Results:**

There was a significant reduction (211 928 fewer tests) in the number of tests performed during the intervention (434 790) compared to before the intervention (646 718). Laboratory test expenditure was $1 663 756.72 USD before the intervention period and $1 105 036.88 USD during the intervention period, demonstrating a cost savings of $558 719.84 USD.

**Conclusion:**

Electronic gatekeeping is a cost-effective intervention for managing medical laboratory expenditures. We recommend that the health sector scale up this intervention nationally.

**What this study adds:**

Using an interrupted time series interval, the authors determined that EGK is a cost-effective intervention for managing medical laboratory expenditures at a tertiary hospital. This study’s findings can promote and contribute to improved laboratory systems and test investigations.

## Introduction

On a global scale, countries report an increase in the cost of healthcare and medical laboratory testing.^1^ In 2020, the United States Department of Health and Human Services reported an increase of $459 million United States dollars (USD) in laboratory test expenditure in 2018 – an upswing from the previous year’s expenditure of $7.1 billion USD. The primary sources of the increased spending were identified as an increase in the number of genetic tests requested, the cessation of discounts for specific chemistry tests, and a change in payment rates.^2^ Similarly, in the United Kingdom, the National Health Service reported an increase in expenditure from £4 billion British pounds to £5 billion British pounds within a five-year period.^3^ Canada has also reported a similar trend in medical laboratory expenditure, with an annual increase of 5.3% attributable to increased laboratory testing based on unnecessary and repeated medical laboratory investigations as well as a hesitancy to develop advanced technological and digital interventions.^4^ In tandem with the increases in medical laboratory expenditures reported by these developed countries, the South Africa National Health Laboratory Service (NHLS) reported a 45% increase between 2010/2011 and 2013/2014, totalling R4.5 billion South African rand.^5^ Laboratory expenditure rose to R7.1 billion in 2016/2017,^6,7,8,9^ R8.2 billion in 2017/2018, and R10.7 billion in 2020/2021.^10,11,12,13^ To address these increasing rates of expenditure by the medical laboratory sector, healthcare settings, specifically in the developed parts of the world, have considered various approaches. Strategies that have been implemented include but are not confined to computerised or electronic order entry systems,^14^ laboratory test profiles,^15^ education^16^ and minimum re-testing intervals.^17^ There is variable evidence of the effectiveness of these strategies as they are effective in some contexts and ineffective in others.^18^ In addition, some hospital settings have implemented a single strategy while others have implemented a multi-pronged approach.

Except for South Africa, most African countries lack published data on medical laboratory expenditures that could facilitate the implementation of cost-effective strategies to manage the demand for laboratory test investigations on the African continent. Furthermore, the published reports in South Africa are insufficient to establish clear patterns to guide the implementation of effective interventions. In the South African public health sector, the NHLS and Department of Health developed a financing mechanism that levies a fee for its services: the fee for service payment model. This model has been identified as a contributor to the escalating costs in the healthcare system and a barrier to integrated care by promoting fragmentation and higher spending.^19^ To overcome this challenge, in 2010, a demand management system known as electronic gatekeeping (EGK) was introduced and piloted at a tertiary academic hospital in the Western Cape. In 2015, the intervention was rolled out in other provinces in the country, including four tertiary academic hospitals in the Eastern Cape. Thus far, only two studies conducted in urban tertiary academic hospitals have attempted to provide evidence of the effectiveness of EGK as a demand management system. One of these studies^20^ concluded that the EGK intervention was an effective method of demand management, while the other study^21^ found the intervention not to have a substantial effect on the way clinicians request laboratory investigations. Given the varying findings from the only two published studies on the effectiveness of EGK as an intervention in South Africa, this study was conducted to evaluate the impact of EGK on laboratory expenditure at a tertiary hospital in the Eastern Cape, South Africa.

## Methods

### Ethical considerations

The Walter Sisulu University Human Research Ethics and Biosafety Committee in South Africa reviewed and approved this study (062/2019). The study only involved the use of secondary data on expenditure, primarily collected electronically by the NHLS and supplied to NMAH. Patient consent was not required and thus waived by the ethics committee because data were secondary and lacked any identifiers linking to any patient. Data protection in place included first obtaining necessary approvals from the ethics committee and hospital management, and ensuring that the data remained anonymous and were only accessed by the researcher.

### Study design

**FIGURE 1:**
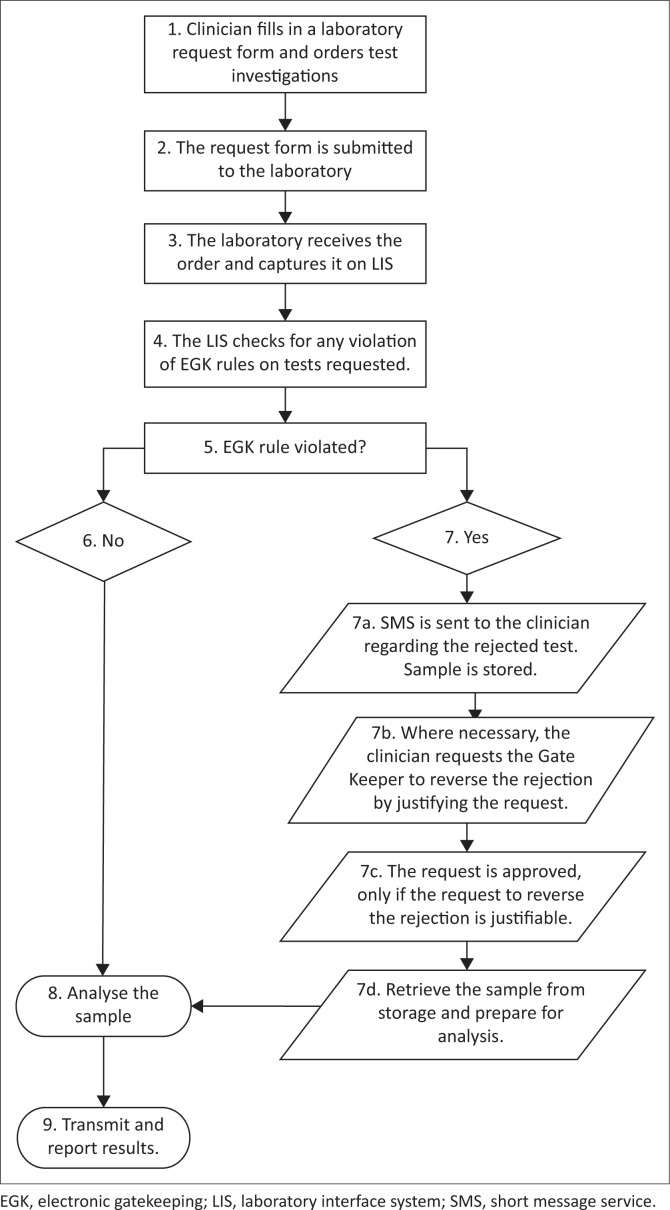
Decision tree applied using the electronic gatekeeping demand management system for the approval of electronic gatekeeping-subjected laboratory tests that were rejected at Nelson Mandela Academic Hospital, South Africa, between 01 June 2015 and 31 May 2017.

This cross-sectional study used an interrupted time series analysis (ITSA) to determine the impact of the EGK intervention on laboratory expenditure, comparing the period before (24 months from 01 June 2013 to 31 May 2015) and during (24 months from 01 June 2015 to 31 May 2017) the EGK intervention. The EGK is a demand management system embedded in a laboratory interface system. The system uses a decision tree as an analytical tool to approve or reject laboratory tests requested by clinicians for patients based on the protocols for patient management devised by hospital medical specialists and consultants^22^ (Figure 1). This decision tree is simple and transparent, representing a series of actions and possible outcomes that can help determine whether a decision should be made and reveal the optimum choice for each scenario.^23^ Before the EGK intervention, there was no other intervention in place to improve laboratory expenditure in the country.

### Study setting

This study was conducted at the Nelson Mandela Academic Hospital (NMAH), which is situated in Mthatha, a small rural town in the OR Tambo District Municipality in the Eastern Cape province of South Africa. In 2019, the district was estimated to have a population of 1 514 306 million.^24^ The NMAH has three academic arms: patient management, medical training and research, with the NHLS pathology laboratory attached to it. The NMAH is the region’s referral hospital, which provides specialist medical services and has a 576-bed capacity with inpatient wards, outpatient clinics and theatres.^25^

### Study data and variables

The study included 28 conveniently sampled laboratory tests that were subjected to EGK. Secondary data on the number, type and costs of tests performed before and during EGK intervention were obtained from NHLS monthly reports, which were generated electronically and provided on spreadsheets using Microsoft Excel 2016 (Microsoft Corporation, Redmond, Washington, United States). These reports are routinely supplied by the NHLS to NMAH to monitor and evaluate laboratory accounts. The variables in the reports include the type of tests, number of tests, expenditure on the tests, and dates the tests were performed (month and year).

### Economic analysis

For this study’s economic evaluation, only the costs of the tests analysed over the four-year period were included in the main analysis. All monetary values were converted from South African rand to USD using a conversion rate of R14.92 to $1.00 USD as per the South African Reserve Bank exchange rate on 17 March 2021.^26^

### Statistical analysis

**FIGURE 2:**
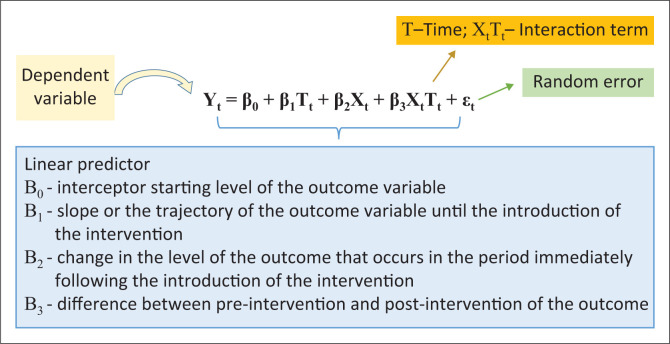
Interrupted time series analysis model equation used to estimate the effects of electronic gatekeeping intervention on expenditure at Nelson Mandela Academic Hospital, South Africa, between 01 June 2015 and 31 May 2017.

Statistical analysis involved the use of an ITSA to analyse changes in expenditure trends. The ITSA, also termed a regression model, is most suited to comparing the impact of interventions before, during and after the event^27,28,29^ (Figure 2). This allows for the evaluation of interventions that have been rolled out at well-defined time points or at well-spaced intervals before, during and after interventions.^30,31^ Statistical analyses were conducted using Stata version 14.2 (StataCorp, College Station, Texas, United States). Newey-West standard errors were reported to account for autocorrelation. We assumed that the outcome variable would not be altered without the EGK intervention, that is, the pre-intervention trend would continue unchanged.

## Results

### Regression analysis on expenditure before and during electronic gatekeeping intervention

Of the 28 tests analysed, 20 tests demonstrated an increasing expenditure trend before the implementation of the intervention, while the remaining eight tests showed a decreasing expenditure trend. These eight tests were phosphorus, cholesterol, triglycerides, high-density lipoprotein, hepatitis A immunoglobulin M, hepatitis B surface antigen, hepatitis B surface antibody, and hepatitis C antibody tests. In the first month of the intervention, 27 of the 28 tests demonstrated a decreasing expenditure trend, with only hepatitis B surface antibody demonstrating an increasing trend. In the months following the first month of the intervention – June 2015 to May 2017 – the trajectory of the expenditure for 64% (18/28) of the tests continued a decreasing trend, whereas the remaining 36% (10/28) continued an increasing trend. These tests were glycated haemoglobin, phosphorus, cholesterol, triglycerides, C-reactive protein, thyroid stimulating hormone, hepatitis A immunoglobulin M, hepatitis B surface antigen, hepatitis B surface antibody, and hepatitis C antibody. For ease of interpretation of the results, all tests presented hereafter are categorised according to their clinical significance.

### Evaluation of trends in expenditure before and during electronic gatekeeping intervention

#### Kidney function tests – Urea and creatinine

The expenditure on kidney function-related tests (creatinine and urea) demonstrated a significant monthly increase prior to the intervention. In the first month of the intervention, the expenditure decreased significantly for creatinine ($856.95 USD reduction, *p* = 0.002) and urea ($817.60 USD reduction, *p* = 0.002). This was followed by an insignificant decreasing trend in monthly expenditure for both tests. After the first month of the intervention, the expenditures for both creatinine ($36.29 USD increase, *p* = 0.007) and urea ($32.11 USD increase, *p* = 0.011) were estimated to increase significantly every month (Online Supplementary Figure 1 and Online Supplementary Table 1).

#### Bone and mineral metabolism-related tests – Calcium, magnesium and phosphorus

Before the intervention, the expenditures on calcium and magnesium tests demonstrated statistically insignificant increases every month, while expenditure on phosphate tests decreased insignificantly. In the first month of the intervention, there was a notable decrease in expenditure on all these three tests, with a statistically significant decrease in expenditure on calcium tests ($667.38 USD decrease, *p* < 0.001) and magnesium tests ($754.78 USD decrease, *p* < 0.001). Expenditure on phosphate tests decreased ($408.63 USD decrease, *p* = 0.178) as well but not significantly. After the first month of the intervention, expenditures on all three tests were estimated to decrease insignificantly every month for calcium ($7.25 USD decrease, *p* = 0.139), magnesium ($4.13 USD decrease, *p* = 0.38) and phosphate ($4.46 USD decrease, *p* = 0.290) (Online Supplementary Figure 2 and Online Supplementary Table 2).

#### Indicator test for long-term glycaemic control – Glycated haemoglobin

There was a statistically insignificant increase in monthly expenditure for glycated haemoglobin tests before the intervention. In the first month of the intervention, a statistically significant increase in expenditure was observed in the first month of the intervention, a statistically significant increase in expenditure was observed ($13.50 USD increase, *p* < 0.001). After the first month of the intervention, the monthly test expenditure was estimated to increase significantly ($13.62 USD increase, *p* = 0.00). (Online Supplementary Table 3 and Online Supplementary Figure 3).

**FIGURE 3:**
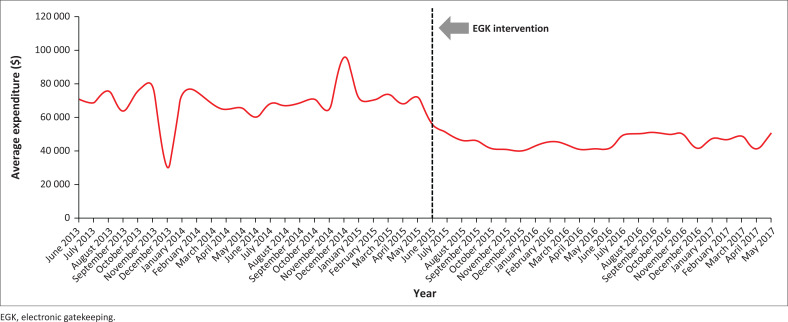
Expenditure trends on tests conducted at the Nelson Mandela Academic Hospital, South Africa, before (01 June 2013 to 31 May 2015) and during (01 June 2015 to 31 May 2017) electronic gatekeeping intervention. The graph shows a clear reduction in expenditure during the electronic gatekeeping intervention period compared to the period before the intervention.

#### Liver function-related tests

Before the intervention, expenditures on total protein and albumin tests increased insignificantly every month. In the first month of the intervention, both test expenditures demonstrated a significant decrease (total protein: $914.47 USD decrease, *p* < 0.001; albumin: $1813.12 USD decrease, *p* < 0.001). After the first month of the intervention, expenditure for total protein tests was estimated to decrease insignificantly ($3.98 USD decrease, *p* = 0.522) and expenditure for albumin was estimated to decrease significantly ($30.71 USD decrease, *p* = 0.026) (Supplementary Figure 4 and Supplementary Table 4).

Total expenditure for total bilirubin and conjugated bilirubin tests increased insignificantly every month before the intervention. In the first month of the intervention, expenditure on both tests decreased significantly (total bilirubin: $1259.99 USD decrease, *p* < 0.001; conjugated bilirubin: $810.57 USD decrease, *p* < 0.001). After the first month of the intervention, both test expenditures were estimated to decrease monthly (total bilirubin: $5.07 USD decrease, *p* = 0.596; conjugated bilirubin: $26.62 USD decrease, *p* = 0.002) (Online Supplementary Figure 5 and Online Supplementary Table 5).

Expenditure on both alanine transaminase and aspartate aminotransferase tests increased insignificantly every month before the intervention. In the first month of the intervention, expenditure for both tests demonstrated a statistically significant decrease (alanine transaminase: $1283.30 USD decrease, *p* < 0.001; aspartate aminotransferase: $1288.06 USD decrease, *p* < 0.001). After the first month of the intervention, the alanine transaminase expenditure increased monthly but not significantly ($2.74 USD increase, *p* = 0.683), whereas the aspartate aminotransferase expenditure decreased significantly ($32.08 USD decrease, *p* = 0.004) (Online Supplementary Figure 6 and Online Supplementary Table 6).

In the first month of the intervention, there were significant decreases in expenditure for both the alkaline phosphatase ($1183.30 USD decrease, *p* < 0.001) and gamma-glutamyl transferase ($1272.27 USD decrease; *p* < 0.001) tests. After the first month of the intervention, the alkaline phosphatase expenditure was estimated to decrease significantly monthly ($37.27 USD decrease, *p* = 0.001) and gamma-glutamyl transferase expenditure was estimated to decrease insignificantly ($3.74 USD decrease, *p* = 0.704) (Online Supplementary Figure 7 and Online Supplementary Table 7).

#### Inflammatory marker-related tests – C-reactive protein

In the first month of the intervention, the C-reactive protein expenditure decreased significantly ($1784.05 USD decrease, *p* < 0.001). However, after the first month of the intervention, the C-reactive protein expenditure was estimated to increase significantly every month ($89.26 USD increase, *p* = 0.000) (Online Supplementary Figure 8 and Online Supplementary Table 8).

#### Lipidaemia-related tests – Cholesterol, triglycerides and high-density lipoprotein

Expenditure on all three lipidaemia-related tests decreased insignificantly every month before the intervention. In the first month of the intervention, all three tests demonstrated significant decreases in expenditure (cholesterol: $116.26 USD decrease, *p* < 0.001; triglycerides: $116.33 USD decrease, *p* = 0.026; high-density lipoprotein: $276.38 USD decrease, *p* < 0.001). After the first month of the intervention, expenditure on cholesterol ($0.92 USD increase, *p* = 0.658) and triglycerides ($0.34 USD increase, *p* = 0.937) were estimated to increase monthly but not significantly. The expenditure for high-density lipoprotein was estimated to decrease insignificantly ($4.55 USD decrease, *p* = 0.093) (Online Supplementary Figure 9 and Online Supplementary Table 9).

#### Prostate cancer-related tests – Prostate-specific antigen

Expenditure for the prostate-specific antigen test increased significantly every month before the intervention. In the first month of the intervention, there was a significant decrease ($278.20 USD decrease, *p* < 0.001) in expenditure for this test. It was estimated that after the first month of the intervention, the monthly expenditure for prostate-specific antigen would increase significantly ($8.05 USD increase, *p* = 0.005) (Online Supplementary Figure 10 and Online Supplementary Table 10).

#### Thyroid function-related tests – Thyroid stimulating hormone, thyroxine and triiodothyronine

We noted insignificant increasing trends in monthly expenditures for the thyroid stimulating hormone and thyroxine tests before the implementation of the intervention. During the same period, the monthly expenditure for triiodothyronine increased significantly. In the first month of the intervention, expenditure decreased significantly for thyroid stimulating hormone ($500.21 USD decrease, *p* = 0.041) and triiodothyronine ($506.62 decrease, *p* < 0.001), whereas for thyroxine, expenditure decreased insignificantly ($352.00 USD decrease, *p* = 0.053). After the first month of the intervention, the thyroid stimulating hormone expenditure was estimated to increase significantly ($26.26 USD increase, *p* = 0.000), whereas the thyroxine expenditure was estimated to decrease insignificantly ($7.06 USD decrease, *p* = 0.114). During the same period, the triiodothyronine expenditure was estimated to decrease significantly ($20.32 USD decrease, *p* = 0.001) (Online Supplementary Figure 11 and Online Supplementary Table 11).

#### Anaemia, leukaemia, and abnormal bleeding-related tests – Full blood count and differential count

Expenditures on anaemia, leukaemia, and abnormal bleeding-related tests increased every month before the intervention. The observed increase was statistically significant for full blood count tests and insignificant for differential count tests. In the first month of the intervention, there were significant reductions in expenditures for both tests (full blood count: $3518.21 USD decrease, *p* < 0.001; and differential count: $5103.73 USD decrease, *p* < 0.001). After the first month of the intervention, both test expenditures were estimated to increase insignificantly (full blood count: $50.89 USD increase, *p* = 0.264; differential count: $3.23 USD increase, *p* = 0.848) (Online Supplementary Figure 12 and Online Supplementary Table 12).

#### Liver inflammation-related tests – hepatitis A glycated haemoglobin, hepatitis B surface antigen, hepatitis B surface antibody and hepatitis C antibody

Before the intervention, monthly expenditure for the HAM and hepatitis C antibody tests demonstrated significant decreasing trends, whereas the expenditure for hepatitis B surface antigen and hepatitis B surface antibody showed insignificant decreasing trends. In the first month of the intervention, the monthly expenditure decreased insignificantly for HAM ($238.47 USD decrease, *p* = 0.673), hepatitis B surface antigen ($177.74 USD decrease, *p* = 0.281) and hepatitis C antibody ($10.58 USD decrease, *p* = 0.776). In the same period, hepatitis B surface antibody expenditure showed a statistically insignificant increase ($52.07 USD increase, *p* = 0.776). After the first month of the intervention, expenditure for HAM, hepatitis B surface antibody, and hepatitis C antibody were estimated to decrease significantly every month (HAM: $14.16 USD decrease, *p* = 0.000; hepatitis B surface antibody: $19.74 USD decrease, *p* = 0.023; hepatitis C antibody: $15.31 USD decrease, *p* = 0.007) (Online Supplementary Figure 13 and Online Supplementary Table 13).

#### Impact of electronic gatekeeping intervention on patient volumes

More tests were conducted before the EGK intervention (646 718) than during the intervention (434 790). Before the intervention, the expenditure trend showed irregular fluctuations, whereas during the intervention, the expenditure trend was somewhat more stable (Figure 3). Total expenditure was also greater before the intervention ($1 663 756.72) than during the intervention ($1 105 036.88). In the period before the intervention, the lowest number of tests conducted per month was 12 258, for which the expenditure was $30 285.50. During the same period, the highest number of tests conducted per month was 36 962, for which the expenditure was $96 054.60. In the intervention period, the lowest number of tests conducted per month was 15 215, for which the expenditure was $41 306.86. During the same period, the highest number of tests conducted was 22 696, for which the expenditure was $55 701.49 (Online Supplementary Table 14).

We also conducted analyses to assess the possible impact of patient volumes on laboratory expenditure. We noted that 348 804 patients were admitted to the facility in the 24 months during the intervention, while slightly more than the 336 283 were admitted in the 24 months before the intervention. Similarly, the outpatient headcount during the 24-month intervention was 339 711, which was slightly more than the 258 267 outpatients received at the facility during the 24 months before the intervention.

## Discussion

Analysis of the findings revealed that the introduction and implementation of EGK at NMAH worked well to manage demand for laboratory tests when compared to the periods when there was no EGK implementation. This is better understood when observing the logistic regression analysis of the test expenditures before, during and after the EGK intervention periods. In the period before the intervention, majority of the tests showed an increase in expenditure patterns. In the period during the intervention, the majority of tests showed a decrease in expenditure patterns, and post intervention, the linear trend assumed that although the overall expenditure will continue to decrease during this period, expenditure may increase for some of the tests that showed a decrease in expenditure during the intervention period.

In the regression analysis of laboratory expenditure in the period before the intervention, 71% (20/28) of the tests analysed demonstrated an increasing expenditure trend, six of which showed a statistically significant increase in expenditure, including creatinine, urea, C-reactive protein, prostate-specific antigen, triiodothyronine and full blood count. The remaining 29% (8/28) of the tests showed a decreasing expenditure trend, with seven of the eight showing a statistically insignificant decrease, including phosphate, cholesterol, triglycerides, high-density protein, hepatitis B surface antigen, hepatitis B surface antigen, hepatitis B surface antibody, and hepatitis C antibody. During the EGK intervention, 96.4% (27/28) of the tests demonstrated decreasing expenditure trends, which were attributed to or associated with the implementation of the intervention. Only one of the 28 tests (3.6%) – hepatitis B surface antigen – demonstrated an increasing expenditure trend during this period.

These findings demonstrate the importance of interventions to curtail unnecessary testing and reduce expenditure. In the months following the intervention, 64% (18/28) of the tests continued a decreasing expenditure trend, while 36% (10/28) showed an increased expenditure trend. It was noted that one of the factors that contributed to the observed change (increase) in expenditure was that over time, clinicians found ways to circumvent the rules of EGK intervention to access the tests they desired. To ensure the robustness of the intervention, this identified vulnerability needs to be addressed by the stakeholders. Further analyses of the expenditure trends *after* the intervention revealed that expenditure was likely to decrease for 57% (16/28) of the tests (25% significantly and 32% insignificantly) and to increase for the remaining 43% (12/28) (21.5% significantly and 21.5% insignificantly). These results and projections highlight the magnitude of unnecessary laboratory testing commonly performed in the absence of an intervention such as EGK.

In a retrospective study conducted at a Cape Town hospital in South Africa to determine the number of chemistry tests rejected by EGK and subsequently restored over a six-month period, it was shown that out of 68 480 tests that were subjected to EGK, only 6.7% were rejected, while 14.7% of these were restored when the clinician made that request.^20^ The authors in that study concluded that the majority of the rejected tests were unnecessary and real cost savings were made, applauding EGK as a simple, effective and sustainable method of demand management. They also showed that the EGK rejections did not have a negative impact on patient care. Our results are in concordance with these findings and demonstrated that EGK is an effective method of reducing unnecessary laboratory test requests to save costs. However, another 22-month retrospective study carried out at a Pretoria hospital in South Africa to determine the number of chemistry tests rejected by EGK found that of the 1 445 782 tests that were subjected to EGK, only 3.33% were rejected, while 1.3% of these were restored when a request was made by a clinician. The authors reported the cost savings as ‘modest’, concluding that EGK does not have a substantial effect on the clinician test requesting pattern.^21^

When we evaluated trends in expenditure and test volumes before and during the intervention, we noted that more money was spent on laboratory investigations before the intervention compared to the intervention period. There were more tests analysed before the intervention compared to during the intervention. It is important to note that in some instances, increases in expenditure for certain tests during the EGK intervention period could also be attributed to inflation. On evaluation of the impact of patient volumes on laboratory expenditure, we noted that there were more patients at the hospital during the intervention period compared to before the intervention. Since test volumes and expenditures remained lower during the intervention compared to the period before the intervention, we concluded that the increase in inpatient and outpatient days during the 24-month period of the EGK intervention did not affect the test volumes or expenditure during this period. The lower test volumes and expenditure may thus be attributed to the implementation of EGK at the facility. We further state that the robust results provided by our estimates through ITSA may provide foundations for public policy decision-making within the South African national health system, especially regarding interventions with significant potential for the reduction of laboratory expenditure related to unnecessary testing.

### Limitations

The study was limited to NMAH and the results may not be representative of other health facilities that might have implemented EGK in South Africa. To the best of our knowledge, this is the first EGK study in South Africa to use an ITSA design to assess the impact of the intervention. As opposed to the other two published reports on EGK effectiveness in South Africa,^20,21^ which only evaluated the costs and effectiveness of the EGK intervention based on rejected tests, our study design had the advantage of assessing trends in longitudinal data and thus revealing pre-intervention trends through regression modelling.^28,29,32^ Given this scarcity of evidence in the study setting and geographical region, as well as the uniqueness of our methodological approach, our findings could not be compared to other studies for similarities or differences. Our study did not also investigate the clinical impact of EGK rejections on patient care.

### Conclusion

By assessing expenditure trends before and during the EGK intervention, we have established that EGK is a cost-saving and effective intervention for managing the demand for laboratory testing. The results obtained in this study highlight the importance of interventions to restrict unnecessary medical laboratory testing, which would reduce wastage and associated costs. This study is unique and has provided strong evidence in support of introducing and implementing EGK in laboratories as a means for the Department of Health to maintain affordable and sustainable costs of laboratory testing.
